# Comparison of Diagnostic Accuracy of Microscopy and Flow Cytometry in Evaluating *N*-Methyl-D-Aspartate Receptor Antibodies in Serum Using a Live Cell-Based Assay

**DOI:** 10.1371/journal.pone.0122037

**Published:** 2015-03-27

**Authors:** Melanie Ramberger, Patrick Peschl, Kathrin Schanda, Regina Irschick, Romana Höftberger, Florian Deisenhammer, Kevin Rostásy, Thomas Berger, Josep Dalmau, Markus Reindl

**Affiliations:** 1 Clinical Department of Neurology, Medical University of Innsbruck, Innsbruck, Austria; 2 Division of Neuroanatomy, Medical University of Innsbruck, Innsbruck, Austria; 3 Institute of Neurology, Medical University of Vienna, Vienna, Austria; 4 Institució Catalana de Recerca i Estudis Avançats (ICREA), IDIBAPS, Hospital Clínic, Barcelona, Spain; 5 Pediatric Neurology, Witten/Herdecke University, Children’s Hospital Datteln, Datteln, Germany; University Hospital-Eppendorf, GERMANY

## Abstract

*N*-methyl-D-aspartate receptor (NMDAR) encephalitis is an autoimmune neurological disease, diagnosed by a specific autoantibody against NMDAR. Antibody testing using commercially available cell-based assays (CBA) or immunohistochemistry on rat brain tissue has proven high specificity and sensitivity. Here we compare an immunofluorescence live CBA to a flow cytometry (FACS) based assay to detect NMDAR antibodies by their binding to the surface of HEK293A cells functionally expressing NMDAR. Both assays were first established using a discovery group of 76 individuals and then validated in a group of 32 patients in a blinded manner. In the CBA, 23 of 23 patients with NMDAR encephalitis were positive for NMDAR antibodies and 0 of 85 controls (32 healthy controls and 53 patients with other neurological diseases), resulting in a sensitivity and specificity of 100% (95% confidence intervals (CI) 85.1–100.0 and 95.7–100.0, respectively). The FACS based assay detected NMDAR antibodies in 20 of 23 patients and in 0 of 85 controls. Therefore, with an equally high specificity (95% CI 95.7–100.0) the sensitivity of the FACS based assay was 87% (95% CI 66.4–97.2). Comparing antibody titers from CBA with delta median fluorescence intensities from FACS showed a high concordance (kappa = 0.943, p<0.0001) and correlation (r = 0.697, p<0.0001). In conclusion, evaluation of the FACS based assay revealed a lower sensitivity and high inter-assay variation, making the CBA a more reliable detection method.

## Introduction

With the identification of antibodies against *N*-methyl-D-aspartate receptors (NMDAR) a new subgroup of autoimmune encephalitis was described in 2007 [1]. The association of prominent psychiatric symptoms in the context of severe encephalitis and an underlying ovarian teratoma initially facilitated the discovery of this disorder [1, 2]. A majority of patients are female and the disease often occurs in childhood or early adolescence [3, 4]. In adults, symptoms are initially psychiatric with insomnia and agitation, followed by dyskinesias, seizures, memory deficits, speech problems and a decrease in the level of consciousness often leading to autonomic instabilities [5–7]. In children, the first symptoms are often seizures or dyskinesias, subsequently progressing to develop the other components of the syndrome. Immunotherapy is effective in most patients, limiting the frequency of relapses and lethality [4]. After the first description of NMDAR antibodies, patients initially presenting with encephalitic and epileptic symptoms of unknown origin became more frequently diagnosed with NMDAR encephalitis [3, 4, 8–14]. The gold standard for the detection of disease specific NMDAR antibodies, which is crucial for the diagnosis of NMDAR encephalitis, comprises testing the immunoreactive binding of serum and cerebrospinal fluid (CSF) samples to fixed and permeabilized NMDAR transfected cells (fixed cell-based assay [CBA]) and immunohistochemistry of frozen sections of rat brain optimized for the detection of antibodies against cell surface or synaptic proteins [5, 15]. Alternatively, CBA using live cells with subsequent fixation can be used to detect autoantibodies against NMDAR [7], although the live CBA was suggested to have a lower sensitivity compared to the fixed CBA [16].

NMDAR are heterotetramers composed of three different NMDAR subunits (NR1-3). Whereas NR1 is ubiquitously present and required for expression of functional NMDAR on the cell surface, distinct NR2(A-D) and NR3(A, B) subunits assemble with NR1 [17]. Hippocampal NMDAR are predominantly composed of NR1 in combination with NR2A and/or NR2B, with an age-dependent shift from NR2B to NR2A [18]. It is now well established that antibodies from NMDAR encephalitis patients are of the immunoglobulin G (IgG) subclass and react with an N-terminal epitope on the NR1 subunit. The binding to NR1 depends on the conformation of the antigen using either live or fixed NMDAR expressing cells, with or without the presence of NR2 subunits [5, 16, 19].

In the present study we compare another live CBA using HEK293A cells expressing NR1/NR2A/NR2B containing functional NMDAR followed by microscopic analysis to a flow cytometry (FACS) based analysis of the test, since the evaluation of cell surface staining by fluorescence microscopy is strongly dependent on the experience of the investigators. Furthermore, a FACS based analysis to detect antibodies to surface antigens would enable quantification of antibody levels over time and also to precisely calculate intrathecal synthesis of the antibodies in those diseases. To assess diagnostic accuracy we evaluate the performance of the two detection methods used.

## Materials and Methods

### Patients

Serum samples from patients and controls were collected in the Clinical Department of Neurology Innsbruck and the Hospital Clínic Barcelona between 2005 and 2013, and stored at -80°C until use. The discovery group (76 individuals from Innsbruck) consisted of seven patients with NMDAR encephalitis, 37 neurological controls (multiple sclerosis n = 33, clinically isolated syndrome n = 3, viral encephalitis n = 1) and 32 healthy controls. The validation group (32 patients from Barcelona) consisted of 16 patients with NMDAR encephalitis and 16 neurological controls (neuromyelitis optica n = 4, multiple sclerosis n = 1, patients with suspected autoimmune encephalitis, including limbic encephalitis, non-focal encephalitis, encephalomyelitis, cerebellar dysfunction, and one patient with hypophysitis n = 11).

Diagnosis of NMDAR encephalitis was based on clinical assessment (new onset of neuropsychiatric symptoms) and demonstration of antibodies in serum or CSF with at least two assays (CBA with fixed cells and tissue immunohistochemistry) as recommended recently [16]. In the discovery group the clinical diagnosis of NMDAR encephalitis diagnosis was confirmed by the presence of NMDAR antibodies in the serum and CSF of patients. One sample was tested in a diagnostic laboratory (Oxford Neuroimmunology Testing Service, Oxford, UK), two samples were tested in our laboratory using a commercially available certified test kit (Euroimmun AG, Lübeck, Germany), and four samples were tested in both laboratories.

In the blinded validation group from Barcelona diagnosis was confirmed by the research center of neuroimmunology (IDIBAPS, Hospital Clínic, University of Barcelona, Spain) using an in-house CBA and tissue immunohistochemistry in CSF and serum samples. Antibody negativity was proven for all control samples of the validation group. All samples of the validation group were blinded by RH and JD. The demographic data of both groups are shown in Table 1. The present study was approved by the Ethical Committee of the Medical University of Innsbruck (study numbers AM3041A and AM4059). All patients and controls gave written informed consent to the study protocol. All samples from the Hospital Clínic Barcelona were deposited in the collection of biological samples named “neuroimmunologia” registered in the biobank of IDIBAPS, Barcelona, Spain. Samples were handled in an anonymized way, thus the Comité Ético de Investigación Clínica of Hospital Clínic de Barcelona accepted to waive the specific written informed consent from the patients or next of kin.

**Table 1:** Demographic data of patients and controls.

**Discovery group (n = 76)**				
	**NMDAR-E**	**NC**	**HC**	**p-value**
Number	7	37	32	
Females	5 (71%)	21 (57%)	27 (84%)	0.045^2^
Age (years)^1^	20 (5–34)	40 (23–69)	43 (27–68)	0.001^3^
CBA NMDAR IgG	7 (100%)	0 (0%)	0 (0%)	<0.0001^2^
FACS NMDAR IgG	6 (86%)	0 (0%)	0 (0%)	<0.0001^2^
**Validation group (n = 32)**				
	**NMDAR-E**	**NC**		**p-value**
Number	16	16		
Females	11 (69%)	9 (56%)		0.716^4^
Age (years)^1^	16 (3–42)	28 (4–70)		0.001^5^
CBA NMDAR IgG	16 (100%)	0 (0%)		<0.0001^4^
FACS NMDAR IgG	14 (87%)	0 (0%)		<0.0001^4^

CBA = cell-based assay. FACS = fluorescence activated cell sorting. HC = healthy controls. NC = neurological controls. NMDAR-E = *N*-methyl-D-aspartate receptor encephalitis.

^1^ Data are shown as median (range), p-value: groups were compared using

^2^ Chi-Square test and

^3^ Kruskal-Wallis test,

^4^ Fisher’s exact test and

^5^ Mann-Whitney *U* test.

### Transient expression of human NMDAR in HEK293A cells and live cell-based immunofluorescence assay (CBA)

Complementary DNA (cDNA) of human (h)GRIN1, NM_000832.5, (Origene, Rockville, MD) was amplified and cloned into the mammalian expression vector Vivid Colors pcDNA 6.2C-EmGFP-GW/TOPO (Life Technologies, Carlsbad, CA), resulting in hGRIN1 C-terminally fused to emerald green fluorescent protein (EmGFP). Correct insert sequence was verified by DNA sequencing (Microsynth, Balgach, Switzerland). Human GRIN2A cDNA (NM_000833.3, expression vector pDEST26) was purchased from Source BioScience (Nottingham, UK). Human GRIN2B cDNA (NM_000834.2) C-terminally fused to GFP (expression vector pCMV6-AC-GFP) was purchased from Origene.

HEK293A cells (ATCC, LGC Standards GmbH, Wesel, Germany) were grown in Dulbecco’s modified Eagle’s medium supplemented with 2 mM L-glutamine (Life Technologies, Art. No. 41965-039), 1 x non-essential amino acids (Life Technologies, Art. No. 11140–050), and 10% fetal calf serum (FCS; Life Technologies, Art. No. 10270-106). For transfection cells were seeded in tissue culture test plates 96F (TPP, Trasadingen, Switzerland, Art. No. 92096) at a density of 2 x 10^4^ cells per 100 μl per well. After 24 hours cells were transfected with three NMDAR subunits hGRIN1-EmGFP, hGRIN2A and hGRIN2B-GFP at a molar ratio of 3:1:1 using FuGENE HD transfection reagent (Promega, Madison, WI, Art. No. E2312) and protected with 30 μM (+)-MK-801 (Sigma-Aldrich, St. Louis, MO, Art. No. M107). Overall efficiency of transfection was determined by flow cytometry (BD Accuri C6; Becton Dickinson, Franklin Lakes, NJ) and transfection rates of NMDAR subunits were determined by antibody staining: cells were fixed with ice cold methanol for ten minutes, blocked at room temperature with 40 μg/ml goat IgG (Sigma-Aldrich, Art. No. I5256) for 15 minutes in phosphate buffered saline/10% heat-inactivated FCS (Sigma-Aldrich, Art. No. F0804; washing buffer, in which all subsequent dilutions were made) and incubated with anti-NR1 (1:500; Millipore, Temecula, CA, Art. No. MAB363), anti-NR2A (1:500; Millipore, Art. No. MAB5216) or anti-NR2B (1:300; Novus Biologicals, Cambridge, UK, Art. No. NB100-74475) antibodies with orbital shaking (200 rpm) at 4°C for one hour. After three washing steps cells were incubated with Alexa Fluor 546 goat anti-mouse IgG antibody (1:1,000; Life Technologies, Art. No. A-11030) for 30 minutes at room temperature without agitation.

For live cell staining, all dilutions were made in washing buffer containing 20 μM (+)-MK-801 (Sigma-Aldrich). Forty-eight hours post transfection live cells were blocked with 40 μg/ml goat IgG (Sigma-Aldrich) for 15 minutes at room temperature, incubated with serum samples at serial dilutions of 1:20, 1:40 and 1:80 for one hour without agitation at 4°C, washed three times and bound antibodies were visualized by incubation with Cy3-conjugated goat anti-human IgG(H+L) antibody (1:300; Jackson ImmunoResearch Laboratory, West Grove, PA, Art. No. 109-166-088) for 30 minutes at room temperature without agitation. For nuclear staining 4’,6-diamidino-2-phenylindole (DAPI; Sigma-Aldrich, Art. No. D8417) was used to exclude dead cells. For this purpose, 0.1 μg/ml DAPI were added after three washing steps. Microscopic examination was done by two independent investigators blinded for any clinical data (MeR, KS and MaR) using a DMI 4000B inverse microscope (Leica, Wetzlar, Germany). Excitation and emission wave lengths to visualize fluorophores were as follows: EmGFP/GFP: 490/15 and 535/35 nm; Cy3: 570/25 and 630/60 nm; DAPI: 400/15 and 460/25 nm. Samples positive for NMDAR antibodies were further serially diluted to assess endpoint antibody titers (1:20, 1:40, 1:80, 1:160 etc.).

In order to visualize the colocalization of NMDAR and serum antibodies, cells were grown on angiogenesis μ-slides (ibidi, Martinsried, Germany, Art. No. 81506), transfected with the three NMDAR subunits hGRIN1-EmGFP, hGRIN2A and hGRIN2B-GFP, and incubated with human serum samples as described above with the exception of DAPI staining, which was omitted. Images were obtained with a TCS SP5 confocal laser scanning microscope (Leica).

### Flow cytometry based assay (FACS)

HEK293A cells were grown in tissue culture test plates 6 (TPP, Art. No. 92006) at a density of 3 x 10^5^ cells per 3 ml per well and transfected with human NMDAR as described above. Unspecific binding of serum antibodies was determined by using HEK293A cells transiently transfected with hCD2-EmGFP fusion protein using hCD2 cDNA cloned into Vivid Colors pcDNA 6.2C-EmGFP-GW/TOPO [20].

Forty-eight hours post transfection cells were detached using trypsin without EDTA (0.25% in PBS; GE Healthcare, Chalfont St Giles, UK, Art. No. L11-002). Cells were incubated with 200 μl trypsin for five minutes at room temperature, resuspended in washing buffer containing 20 μM (+)-MK-801 (Sigma-Aldrich), centrifuged at 500 g for five minutes, and stained as described above with the following modifications: all incubation steps were performed at room temperature with orbital shaking at 200 rpm. Washing steps were performed by repeated centrifugation (3 x) at 500 g for five minutes and resuspension of the pellets. Duplicates of serum samples at a dilution of 1:100 were used at a cell density of 2 x 10^5^ cells per 200 μl. Bound serum antibodies were detected by allophycocyanin (APC)-conjugated AffiniPure goat anti-human IgG antibody (1:100; Jackson ImmunoResearch Laboratory, Art. No. 109-136-088). Cells were incubated with 100 μl washing buffer containing 7-amino-actinomycin D (7-AAD; 1:30; Becton Dickinson, Art. No. 559925) to exclude dead cells for ten minutes at room temperature and analyzed on a BD Accuri C6 flow cytometer.

In this manner, a maximum of 22 samples were analyzed in parallel (average 14 samples per analysis). Therefore, not all samples, neither of the discovery nor of the validation group, could be analyzed with the same batch of transfected and trypsinized cells. Consequently, considering the inter-assay variation, when interpreting data from different analysis batches, is of importance. For reanalysis of samples to compare serum dilutions of 1:100 and 1:20 it was taken care that different dilutions of the same sample were analyzed in the very same experiment.

NMDAR and CD2 expressing cells were detected in the green FL-1 channel, dead cells in the red FL-3 channel and antibody binding was measured in the red FL-4 channel. Ten thousand (Em)GFP^pos^ 7-AAD^neg^ cells were acquired for each sample. Antibody bound to cell surface resulted in a shift to the right on the x-axis in FL-4. Median fluorescence intensity (MFI) from CD2 transfected cells was subtracted from MFI of NMDAR expressing cells (ΔMFI). Healthy controls and patients with other neurological diseases were used to calculate the cut-off ΔMFI.

### Statistical analyses

Statistical analyses were done using IBM SPSS software (release 21.0, IBM, Armonk, NY) or GraphPad Prism 6 (GraphPad, San Diego, CA). Between-group comparisons were performed with Kruskal-Wallis test, Dunn’s multiple comparison post-hoc test, Mann-Whitney *U* test, Fisher’s exact test and Chi-square test. Correlation of parameters was analyzed with Spearman’s non-parametric correlation. Receiver operating characteristic (ROC) curve analysis was used to determine cut-off ΔMFI (FACS). Kappa statistics was used to assess the concordance between the two testing methods. Statistical significance was defined as two-sided p-value<0.05 and Bonferroni corrections were applied for multiple comparisons when appropriate.

## Results

### Expression of functional NMDAR

Expression of NMDAR subunits NR1, NR2A and NR2B was verified by staining with antibodies specific for the respective subunit (S1 Fig). We found an optimal distribution of each subunit by using a molar ratio of NR1-EmGFP:2A:2B-GFP of 3:1:1. The overall transfection efficiencies as detected by flow cytometry were 84±7% and 93±6% for NMDAR-(Em)GFP and CD2-EmGFP, respectively. Survival and transfection rates of NMDAR overexpressing cells increased in a dose-dependent manner in the presence of the uncompetitive NMDAR antagonist (+)-MK-801, indicating the presence of functional NMDAR (S2 Fig).

### Detection of NMDAR antibodies in the discovery group

**Fig 1:**
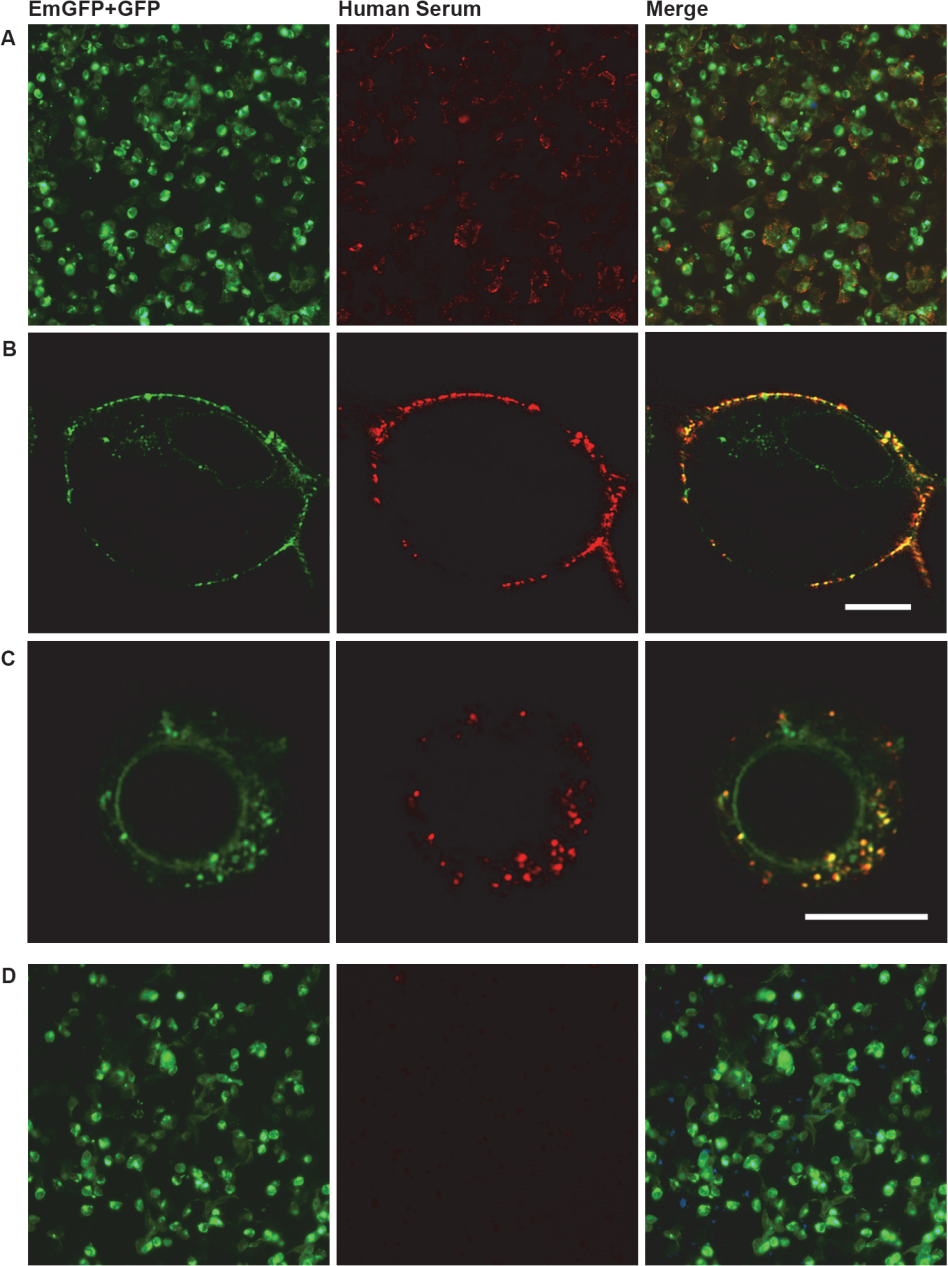
Immunofluorescence CBA with HEK293A cells transiently overexpressing functional NMDAR tagged with green fluorescent proteins. Staining pattern with NMDAR antibody positive (A-C) and negative (D) serum. HEK293A cells were transiently transfected to overexpress EmGFP-tagged NR1, NR2A and GFP-tagged NR2B, incubated with diluted human serum and NMDAR antibodies were visualized by a Cy3-conjugated secondary antibody and counter-stained with DAPI to detect dead cells (left column: green fluorescence/EmGFP+GFP; middle column: red fluorescence/Cy3; right column: overlay of EmGFP/GFP, Cy3 and DAPI (A+D)). (B)+(C) Images show colocalization of NMDAR and serum NMDAR antibodies at high magnification (scale bars: 10 μm). (B) NMDAR antibodies bound to surface of cells. (C) Bound NMDAR antibodies internalized by the cells. CBA = cell-based assay. DAPI = 4’,6-diamidino-2-phenylindole. (Em)GFP = (emerald) green fluorescent protein. NMDAR = *N*-methyl-D-aspartate receptor.

Fig 1 shows the typical antigen distribution of HEK293A cells overexpressing NMDAR tagged with green fluorescent proteins and the staining pattern with serum IgG of an NMDAR encephalitis patient at low magnification and higher magnification using a confocal microscope. It clearly shows the colocalization of membrane-associated NMDAR with serum antibodies of the patient but no colocalization with intracellular NMDAR probably residing within the endoplasmic reticulum (Fig 1B). Internalization of NMDAR in response to antibody binding observed in some but not all cells in the live CBA is shown in Fig 1C.

**Fig 2:**
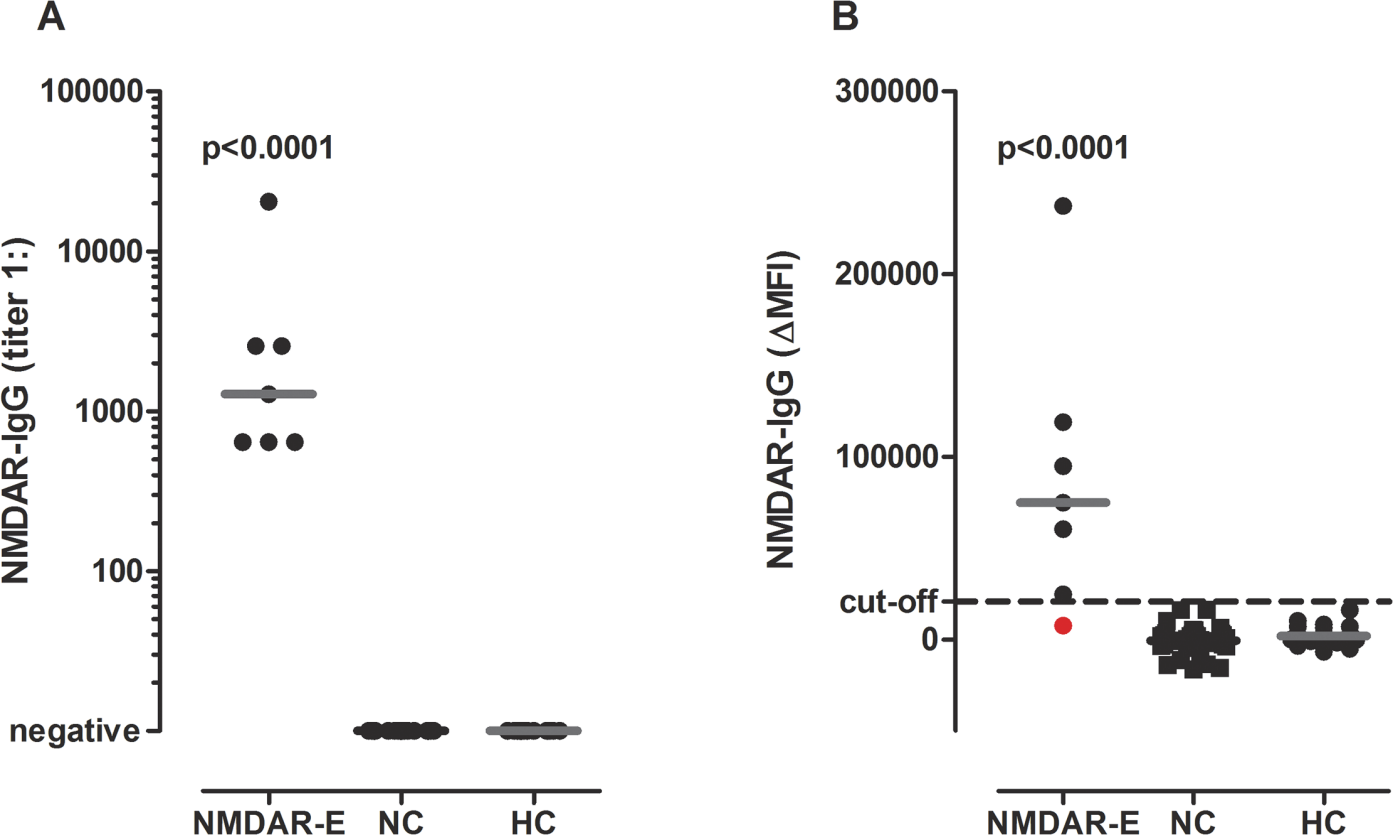
NMDAR IgG antibody titers and ΔMFI values in the discovery group. (A) Using the CBA serum NMDAR IgG antibodies were exclusively detected in serum samples of patients with NMDAR encephalitis, but not in neurological and healthy controls. (B) In the FACS assay serum NMDAR IgG ΔMFI levels were higher in patients with NMDAR encephalitis than in neurological and healthy controls, but one serum positive for NMDAR antibodies was missed with this method (shown in red). The cut-off ΔMFI value of 20,700 is indicated by a dashed horizontal line. Antibody titers and ΔMFI values were compared using a non-parametric test (Kruskal Wallis test) and overall p-values are shown in the graphs. Medians are indicated by horizontal bars. CBA = cell-based assay. ΔMFI = delta median fluorescence intensity. FACS = fluorescence activated cell sorting. HC = healthy controls. NC = neurological controls. NMDAR-E = *N*-methyl-D-aspartate receptor encephalitis.

With the CBA, in the discovery group NMDAR antibodies were detected in 7/7 (100%) patients with NMDAR encephalitis, 0/37 (0%) neurological controls and 0/32 (0%) healthy controls (Table 1). Sensitivity and specificity of the CBA were 100% (95% confidence intervals (CI) 59.0–100.0 and 94.8–100.0, respectively). Antibody titers in NMDAR encephalitis patients ranged from 1:640 to 1:20,480 (median 1:1,280) (Fig 2A).

**Fig 3:**
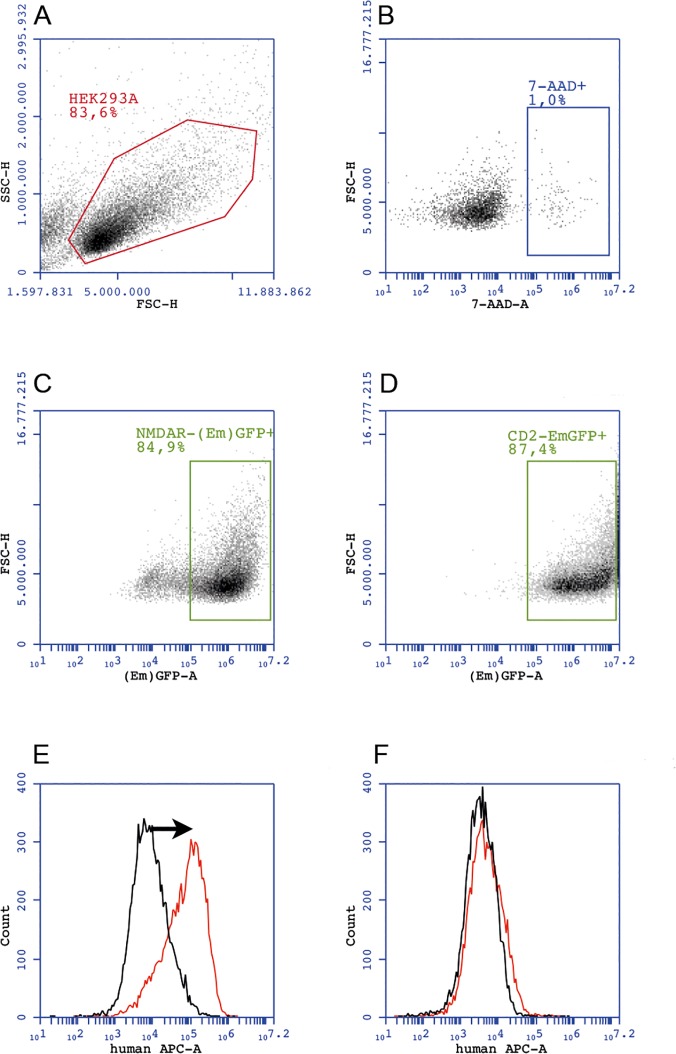
Gating and analysis strategy for NMDAR and CD2 expressing HEK293A cells for FACS based analysis. (A) Gating of the HEK293A main cell population. (B) Exclusion of 7-AAD^pos^ cells. (C)+(D) Gating of (Em)GFP^pos^7-AAD^neg^ NMDAR and CD2 expressing cells, respectively. The green (“(Em)GFP-A”) channel detects both, EmGFP and GFP-tagged proteins. Binding of patient‘s antibodies to NMDAR results in a difference (arrow) of the APC signal obtained with NMDAR-transfected cells (red line) when compared to the APC signal of CD2-transfected cells (black line), which results in ΔMFI (E). This difference is absent in the serum of a healthy control (F). 7-AAD-A = 7-amino-actinomycin D (area). APC-A = allophycocyanin (area). (Em)GFP-A = (emerald) green fluorescent protein (area). ΔMFI = delta median fluorescence intensity. FACS = fluorescence activated cell sorting. FSC-H = forward scatter (height). NMDAR = *N*-methyl-D-aspartate receptor. SSC-H = side scatter (height).

For the FACS based assay, gating and analysis strategy for NMDAR-(Em)GFP and CD2-EmGFP expressing cells is shown in Fig 3. In the discovery group the ΔMFI was significantly higher in NMDAR patients (median 74,938, range 7,681 to 237,432) compared to neurological controls (median -401, range -16,158 to 16,646) and healthy controls (median 1,076, range -6,701 to 16,269; Fig 2B). Using ROC analysis a cut-off ΔMFI value of 20,700 was determined (area under the curve 0.988, p<0.0001). NMDAR antibodies were detected in 6/7 (86%) NMDAR encephalitis patients, 0/37 (0%) neurological and 0/32 (0%) healthy controls (Table 1). Therefore, with a specificity of 100% (95% CI 94.8–100.0) the FACS based assay had a sensitivity of 86% (95% CI 42.1–99.6). Intra- and inter-assay variations (coefficient of variation) were 6% and 22–25%, respectively.

### Detection of NMDAR antibodies in the validation group

**Fig 4:**
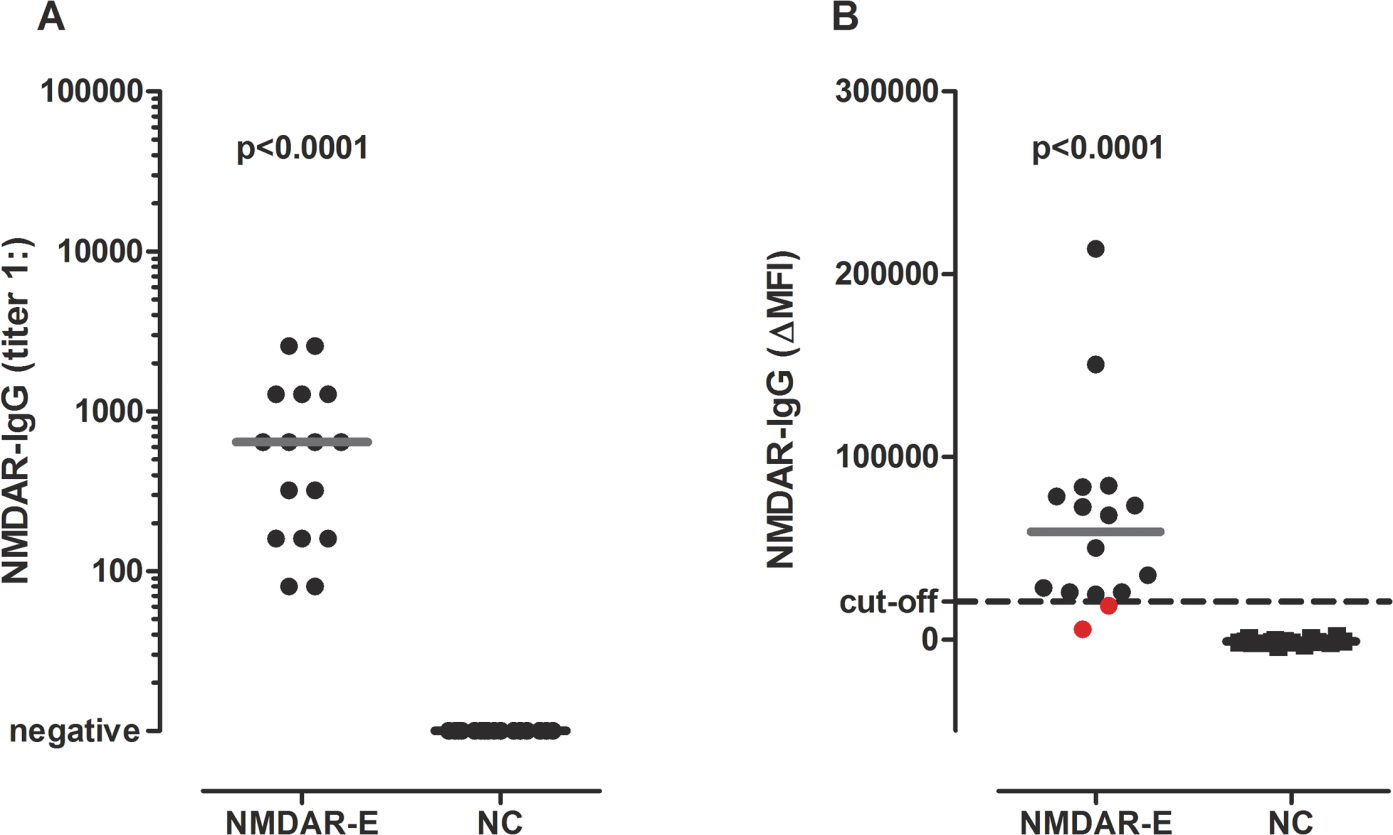
NMDAR IgG antibody titers and ΔMFI values in the validation group. (A) With the CBA all sera of NMDAR encephalitis patients were positive for NMDAR IgG antibodies, but none of the neurological controls. (B) Using the FACS assay serum NMDAR IgG ΔMFI levels were higher in patients with NMDAR encephalitis than in neurological controls, but again two sera positive for NMDAR antibodies were missed with this method (shown in red). The cut-off ΔMFI value of 20,700 as determined in the discovery group is indicated by a dashed horizontal line. Antibody titers and ΔMFI values were compared using a non-parametric test (Mann-Whitney *U* test) and overall p-values are shown in the graphs. Medians are indicated by horizontal bars. CBA = cell-based assay. ΔMFI = delta median fluorescence intensity. FACS = fluorescence activated cell sorting. NC = neurological controls. NMDAR-E = *N*-methyl-D-aspartate receptor encephalitis.

In a next step the CBA was applied to 32 blinded samples of the validation group from Barcelona. All 16 patients with NMDAR encephalitis were positive for NMDAR antibodies and all 16 neurological controls were seronegative (Table 1). Antibody titers in NMDAR encephalitis patients ranged from 1:80 to 1:2,560 (median 1:640) (Fig 4A). Thus, the sensitivity and specificity of the CBA of 100% were confirmed in these blinded samples (95% CI 79.4–100.0).

Likewise, the FACS assay was applied to 32 blinded samples of the validation group from Barcelona. 14/16 patients with NMDAR encephalitis (87%) were positive for NMDAR antibodies using the cut-off value determined in the discovery group and all 16 neurological controls were seronegative (Table 1). The ΔMFI was significantly higher in NMDAR patients (median 59,085, range 5,784 to 213,910) compared to neurological controls (median -1,239, range -3,751 to 2,169, Fig 4B). Thus, the sensitivity and specificity of the FACS assay were equally high in the validation group (95% CI 61.7–98.5 and 79.4–100.0, respectively) as in the discovery group.

### Comparison of CBA and FACS

**Fig 5:**
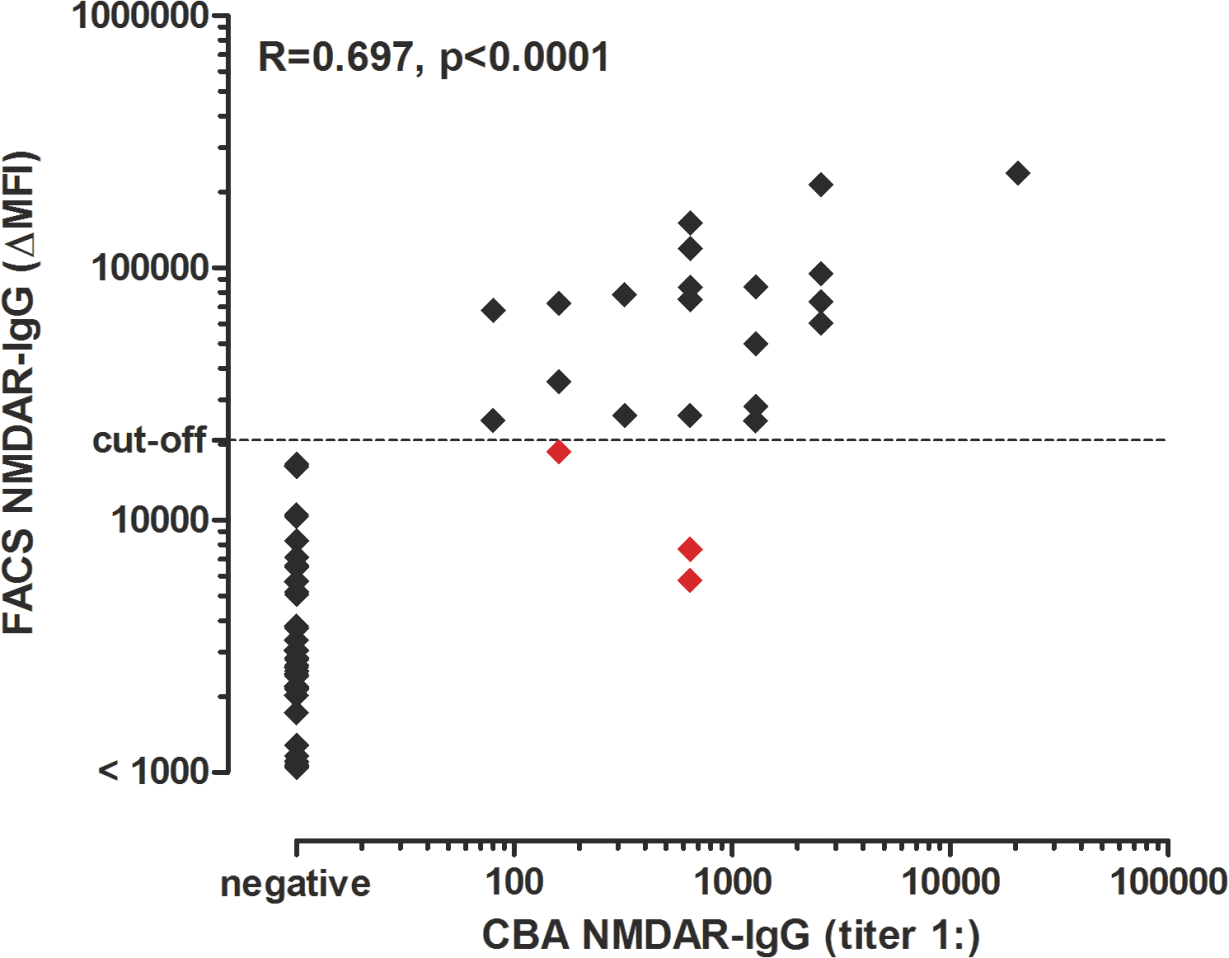
Correlation of NMDAR IgG titers and ΔMFI values determined by CBA and FACS assays. The cut-off value (20,700 ΔMFI, determined by the FACS assay) is indicated by the dashed horizontal line. Correlation of antibody titers and ΔMFI values were calculated using non-parametric Spearman correlation. Correlation coefficient (R) and the p-value are shown in the graph. False negative samples in the FACS assay are depicted in red. In total, 49 samples had a ΔMFI value <1,000, which were all negative in the CBA. CBA = cell-based assay. ΔMFI = delta median fluorescence intensity. FACS = fluorescence activated cell sorting. NMDAR = *N*-methyl-D-aspartate receptor.

The concordance kappa value between CBA and FACS was 0.943 (p<0.0001). 85 samples were seronegative and 20 samples were seropositive with both methods. Three samples were seropositive in the CBA, but seronegative in the FACS assay. Correlation of antibody titers of the CBA with ΔMFI obtained by FACS based analysis was 0.697 (Spearman’s ρ; p<0.0001; Fig 5).

To elucidate why three positive samples could not be detected in the FACS assay, we compared ΔMFI and MFI values resulting from IgG binding to NMDAR and CD2 transfected cells alone. Whereas ΔMFI and MFI values obtained by binding of IgG to NMDAR transfected cells were significantly (p<0.01) lower in false negative samples, MFI obtained by binding of IgG to CD2 transfected cells did not differ between the groups (S3 Fig). Therefore, missing of positive samples cannot be attributed to high background fluorescence intensity, since it was comparable across all samples.

We next analyzed the distribution of NMDAR-(Em)GFP overexpressing HEK293A cell populations resulting from antibody binding of an NMDAR-IgG positive serum. Two distinct EmGFP/GFP positive cell populations were observed in NMDAR antibody positive samples which differed mainly in their size and APC fluorescence signal (S4 Fig). We concluded that those cell populations most likely represent two different cell types: large cells with antibodies bound to the cell surface; and smaller cells with internalized NMDAR in response to antibody binding and/or cells retaining NMDAR in the endoplasmic reticulum, which can also be seen in Fig 1. Comparison of EmGFP/GFP and APC fluorescence intensities resulting from the FACS based assay of human serum samples with different amounts of NMDAR antibodies revealed that the shift to a positive APC signal is not distinct enough in a false negative sample, when using the very same batch of transfected and trypsinized cells (S5 Fig).

### Repeat FACS analysis of a subsample using a lower serum dilution

**Fig 6:**
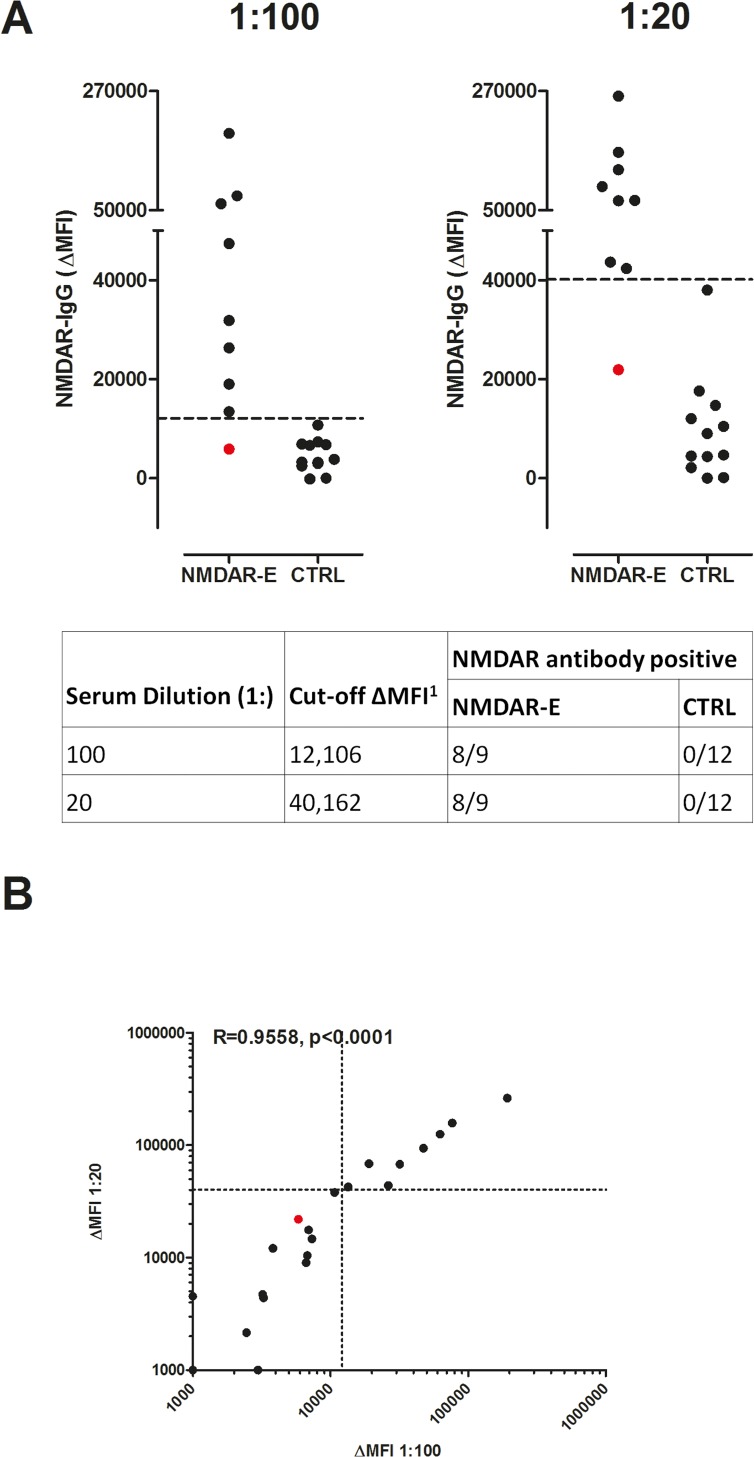
NMDAR antibody ΔMFI at different serum dilutions in NMDAR antibody positive and negative sera. NMDAR antibody positive (n = 9) and negative (n = 12) serum samples have been determined by CBA. (A) Serum dilutions of 1:100 and 1:20 are shown. Respective cut-off ΔMFI values are indicated by dashed horizontal lines. The table shows cut-off ΔMFI and numbers of samples tested positive for NMDAR antibodies by the FACS assay at different serum dilutions. (B) Correlation of ΔMFI obtained by using 1:100 and 1:20 dilution in the re-evaluation group of NMDAR positive samples in the CBA. Respective cut-off values are indicated by dashed lines. The one false negative sample at both dilutions is shown in red. For a better graphical presentation, ΔMFI values below 1,000 were set to 1,000. Correlation of exact ΔMFI values were calculated using non-parametric Spearman correlation. Correlation coefficient (R) and the p-value are shown in the graph. Here, the cut-off at the 1:100 dilution differs from the original cut-off (20,700), since a different batch of cells was used for analysis of this subsample. ^1^ Data were analyzed using ROC analysis. CBA = cell-based assay. CTRL = control sample. ΔMFI = delta median fluorescence intensity. NMDAR-E = *N*-methyl-D-aspartate receptor encephalitis. ROC = receiver operating characteristic.

Aiming to increase the sensitivity by using a lower serum dilution, we reanalyzed 21 samples, including nine positive (six and three from the discovery and validation group, respectively) and 12 negative (each six healthy and neurological controls from the discovery group) for NMDAR antibodies, and compared the previously used 1:100 dilution to a dilution of 1:20. For this comparison, we focused on samples that were false negative or close to the cut-off value during the initial antibody testing with the FACS assay. Using either dilution 8/9 (89%) NMDAR antibody positive and 0/12 (0%) antibody negative samples were detected by the FACS assay. Sensitivity and specificity of both dilutions were therefore comparable to previously obtained results. Interestingly, the cut-off ΔMFI was lower with this set of experiments using the 1:100 dilution compared to previously obtained results (Fig 6), underlining the high inter-assay variation of the FACS based assay. Correlation of ΔMFI at both dilutions was 0.9558 (Spearman’s ρ; p<0.0001; Fig 6B).

Analysis of the re-evaluation group further demonstrated the high variability of the testing system. The inter-assay variation after including new data from the re-evaluation group increased considerably with coefficients of variation of up to 36%. The variability was not correlated with CBA titers (R = 0.3024; Spearman’s ρ; p = 0.4306; S6 Fig).

## Discussion

Although NMDAR encephalitis is considered a rare disease, there is an increasing number of studies identifying this disorder [6, 8, 11–14]. The exact frequency is unknown, but several recent studies with large series of patients [4, 6] and studies focusing on the causes of encephalitis [21, 22] suggest this disorder to be the second most common autoimmune encephalitis after acute disseminated encephalomyelitis, and at some institutions it is more frequent than any encephalitis of viral origin in young patients. Therefore, this form of encephalitis is likely to be underdiagnosed, and there is an increasing need for the availability of antibody testing.

In the present study we compared a live CBA with FACS based analysis to detect serum autoantibodies binding to NMDAR. Sensitivities were high in both testing methods, although we found a higher sensitivity in the CBA (100%) compared to the FACS based analysis (87%). Using a lower serum dilution did not increase the sensitivity of the FACS assay and revealed that the cut-off ΔMFI was variable in a different batch of experiments, further demonstrating a high inter-assay variation. Whereas some samples yielded reproducibly low (and others high) results, even when comparing results from different batches of experiments, others showed a very high inter-assay variability, suggesting that trypsinization might destroy the epitope recognized by some sera but not others. In general, although the inter-assay variation was already high when using the same batch of cells for analysis, it further increased (25 to 36%) when including the same samples analyzed with another batch of cells used for transfection. It is therefore recommended to set a new ΔMFI in every experiment for future attempts to improve a FACS based analysis for detection of surface antigens, which can be a logistical challenge. In our analysis this became even more evident, when two sera that were false negative in the original analysis would have been positive in the second. Consequently, by regularly setting new cut-offs, the sensitivity could possibly be improved, but as one sample was still missed, the CBA would still have yielded a better sensitivity.

False negative samples showed already low NMDAR specific signals rather than high background fluorescence. Although overall we observed a high correlation between CBA titers and ΔMFI, false negative samples did not necessarily have a low titer in the CBA. Therefore it is not likely that the fluorescence signal is too weak to be detected by the flow cytometer. Rather, cells expressing fluorescently labeled NMDAR but partly retaining the receptors intracellularly might accumulate and decrease the relative number of cells expressing NMDAR on their surface, leading to a low signal of surface bound IgG, resulting in the observed lower sensitivity compared to the CBA. As can be seen in visual inspections, the frequency of cells expressing NMDAR on the surface varies, which could also be a source of high inter-assay variation in the FACS based assay. The use of fixed and permeabilized cells would make intracellular epitopes accessible, but then it is not possible to exclude dead cells any more, which could lead to unspecific binding, possibly resulting in a lower specificity.

Both methods are based on the expression of functional NMDAR in HEK293A cells, differing only in the detection of secondary antibody signals. A FACS based analysis would have several advantages. Signal strength correlates with antibody titers and is therefore a quantitative method without the need for analyzing serial dilutions. In contrast to CBA, FACS based analysis does not rely on the experience of the investigators. Furthermore, once a sample has been analyzed, original data can always be reevaluated. However, since the sensitivity of the FACS based analysis was lower (87%), and the inter-assay variation was high, this method is currently not reliable for routine antibody testing. Moreover, FACS based analysis has limitations for the study of CSF samples, which in some patients is crucial for antibody detection. Future studies to improve sensitivity and reproducibility of the FACS based analysis should aim to use non-adherent cells to avoid differential destruction of epitopes by trypsinization. A further challenge will be to adjust cut-offs in every new experiment, e.g. by using internal reference samples or by readjusting the cut-off in every experiment using control sera.

Hippocampal NMDAR form tetramers with two NR1 and two NR2 subunits, with an age-dependent shift from NR2B to NR2A [18]. In contrast to existing testing methods using either transfected cells with only NR1 [23, 24] or in combination with NR2B [7, 16] we used NMDAR containing both NR2A and NR2B, aiming to increase the density of functional NMDAR expressed on the surface of HEK293A cells and not to miss any NMDAR antibodies due to age-dependent changes in subunit composition. NMDAR encephalitis is associated with antibodies recognizing a well-defined epitope on the extracellular region of the NR1 subunit of NMDAR [5, 16, 19]. In contrast, antibodies to the NR2A or NR2B subunits react with a linear epitope and their significance is unclear. In our testing methods, we used live cells expressing functional NMDAR without disruption of the native conformation. Since NR2A and NR2B cannot be expressed on the cell surface without the presence of NR1 [25, 26], we did not find any antibodies recognizing either NR2A or NR2B alone with this setting, neither in NMDAR encephalitis patients nor in controls. Moreover, with our assay we were able to avoid unspecific antibody binding to dead cells. This is not possible in assays where cells are fixed before [5, 15] or after [7, 16] serum (or CSF) incubation as it is done in other labs. In contrast to others that used NMDAR and EGFP co-transfected cells [7] we used NMDAR subunits directly fused to EmGFP (NR1) or GFP (NR2B) for transfection of cells, which enabled us to truly colocalize NMDAR with bound antibodies. Through the combination of using live cells and NMDAR directly linked to a fluorophore we could even visualize the internalization of NMDAR which is known to occur in response to NMDAR antibodies by applying patients’ antibodies to cultured neurons [27] or by intraventricular infusion of patients’ antibodies into mice [28]. And finally, to our knowledge this is the first live CBA that includes protection of the cells by (+)-MK-801 against excitotoxicity throughout the staining procedure, which we found is crucial for not losing living cells that bind NMDAR antibodies to their surface, particularly in the FACS based assay. This might also explain the discrepancy in sensitivity of our test compared to a lower sensitivity in live CBA found by others [16].

In the present study we focused on the establishment of serologic antibody testing methods since the availability of CSF was limited, the collection of CSF is invasive, and serum testing often produces more background than CSF testing. A study investigating the largest known cohort of NMDAR encephalitis patients [4] found a better correlation of CSF antibody titer with disease activity, and CSF was suggested to be more sensitive than serum [29]. Future investigations should aim to optimize the FACS based analysis to provide more reliable results, even when only low CSF volumes are available.

Controversial data exist regarding NMDAR antibody levels in serum and their clinical relevance [30], further underlined by a recent study using a live CBA with fixation after serum incubation that showed serum positivity in 23% of patients with an unlikely autoimmune syndrome, as well as CSF negativity in some cases considered with definite NMDAR encephalitis [31]. In contrast to their live CBA, here we used endpoint titration instead of a visual scoring system. Furthermore, we found that protection of the NMDAR overexpressing cells is necessary throughout the assay to assure their survival, which is of particular importance when assessing undiluted CSF. One limitation of our assay is that we used only sera of NMDAR encephalitis patients that had a definite clinical diagnosis partly based on their seropositivity to assess specificity and sensitivity. This limitation is in part caused by the fact that using previously established criteria that confer high specificity and sensitivity [16], some of the authors (RH and JD) never encountered patients with NMDAR encephalitis without antibodies in the CSF, or patients with serum NMDAR antibodies and an unlikely autoimmune disorder. It is therefore even more important to launch a multicenter study to compare results of different laboratories that conduct NMDAR antibody testing.

In conclusion, we compared two highly sensitive serologic testing methods to detect autoantibodies to NMDAR. In our experience both methods had a high specificity, whereas the sensitivity of the immunofluorescence CBA was higher than that of the FACS assay. This might be especially important for the analysis of CSF antibodies, since they are crucial for the diagnosis of NMDAR encephalitis, and a particularly high sensitivity is needed due to the low amounts of immunoglobulins that can be present in CSF.

## Supporting Information

S1 Fig Staining of NMDAR overexpressing HEK293A cells with antibodies against NMDAR subunits. Cells stained with antibodies against NR1 (A), NR2A (B), and NR2B (C) are shown, respectively.(Em)GFP = (emerald) green fluorescent protein. NMDAR = *N*-methyl-D-aspartate receptor.
(TIF)

S2 Fig Survival and transfection rates of NMDAR-(Em)GFP overexpressing HEK293A cells 48 h post transfection. Dead cells are 7-AAD^pos^, NMDAR expressing cells EmGFP/GFP^pos^. Means of two experiments are shown, bars indicate standard deviation.7-AAD = 7-amino-actinomycin D. (Em)GFP = (emerald) green fluorescent protein. NMDAR = *N*-methyl-D-aspartate receptor.
(TIF)

S3 Fig ΔMFI and MFI obtained by FACS analysis in NMDAR-IgG positive samples as determined by CBA. CBA+FACS+ show samples where NMDAR antibodies were detected with both methods, CBA+FACS- represent samples that were positive in the CBA, but (false) negative in the FACS. (A) ΔMFI (NMDAR-CD2 IgG). (B) MFI of IgG binding to NMDAR only. (C) MFI IgG binding to CD2 only (note that the scale of the y-axis has changed). Medians are indicated by horizontal bars. ΔMFI and MFI values were compared using a non-parametric test (Mann-Whitney *U* test). **p<0.01 CBA = cell-based assay. (Δ)MFI = (delta) median fluorescence intensity. FACS = fluorescence activated cell sorting. NMDAR = *N*-methyl-D-aspartate receptor. ns = not significant.
(TIF)

S4 Fig APC fluorescence according to cell size of an NMDAR-IgG positive (A) and negative (B) sample. Left column: gating of (Em)GFP-positive NMDAR expressing HEK293A cells (excluding dead cells) to discriminate small (Q1-UL) and large (Q1-UR) cells. Middle column: relative APC fluorescence signal of small cells (Q1-UL). A second population with lower APC fluorescence signal is highlighted in blue (P1). Right column: relative APC fluorescence signal of large cells (Q1-UR). P1 decreased from 51.2% to 19.1% in the NMDAR-IgG positive sample. Overall MFI values are shown in the respective graphs. APC-A = allophycocyanin (area). (Em)GFP-A = (emerald) green fluorescent protein (area). FSC-A = forward scatter (area). MFI = median fluorescence intensity. NMDAR = *N*-methyl-D-aspartate receptor. Q1-UL/R = upper left/right quadrant.
(TIF)

S5 Fig Correlation of (Em)GFP and APC fluorescence signal in the FACS based assay. NMDAR transfected HEK293A cells (EmGFP/GFP positive) were incubated with human serum negative for NMDAR antibodies (A), or human serum high (B; CBA titer 1:20,480) and medium (C; CBA titer 1:640) positive for NMDAR antibodies which were detected by an APC-conjugated secondary antibody. The population within the upper right quadrant ((Em)GFP^pos^APC^pos^) represents the cell population expressing NMDAR with bound NMDAR antibodies. (D) shows the cells incubated with a serum negative in the FACS based assay, but positive in the CBA (1:640). Consider that positivity was not determined by the percentage of double positive cells, but the ΔMFI (A: -1,835; B: 290,060; C: 75,976; D: 8,160). APC-A = allophycocyanin (area). CBA = cell-based assay. ΔMFI = delta median fluorescence intensity. (Em)GFP(-A) = (emerald) green fluorescent protein (area). FACS = fluorescence activated cell sorting. NMDAR = *N*-methyl-D-aspartate receptor.
(TIF)

S6 Fig Inter-assay variation of ΔMFI obtained from two independent analysis batches. (A) Individual ΔMFI variability of the nine samples positive for NMDAR antibodies in the CBA. Means are shown as horizontal lines, standard deviations are indicated by grey bars. (B) Correlation of individual ΔMFI variability (CV) and respective NMDAR-IgG titers in the CBA. The correlation was calculated using non-parametric Spearman correlation. Correlation coefficient (R) and the p-value are shown in the graph. Symbols represent matching samples in (A) and (B). Sample Nos. 6 and 7 (A) have the same CV and NMDAR-IgG titer (B). CBA = cell-based assay. CV = coefficient of variation. ΔMFI = delta median fluorescence intensity. NMDAR = *N*-methyl-D-aspartate receptor.
(TIF)

## References

[1] Dalmau J, Tuzun E, Wu HY, Masjuan J, Rossi JE, Voloschin A, et al Paraneoplastic anti-N-methyl-D-aspartate receptor encephalitis associated with ovarian teratoma. Ann Neurol. 2007;61(1):25–36. 17262855 10.1002/ana.21050 PMC2430743

[2] Vitaliani R, Mason W, Ances B, Zwerdling T, Jiang Z, Dalmau J. Paraneoplastic encephalitis, psychiatric symptoms, and hypoventilation in ovarian teratoma. Ann Neurol. 2005;58(4):594–604. 16178029 10.1002/ana.20614 PMC2245881

[3] Florance NR, Davis RL, Lam C, Szperka C, Zhou L, Ahmad S, et al Anti-N-methyl-D-aspartate receptor (NMDAR) encephalitis in children and adolescents. Ann Neurol. 2009;66(1):11–8. 10.1002/ana.21756 19670433 PMC2826225

[4] Titulaer MJ, McCracken L, Gabilondo I, Armangue T, Glaser C, Iizuka T, et al Treatment and prognostic factors for long-term outcome in patients with anti-NMDA receptor encephalitis: an observational cohort study. Lancet Neurol. 2013;12(2):157–65. 10.1016/S1474-4422(12)70310-1 23290630 PMC3563251

[5] Dalmau J, Gleichman AJ, Hughes EG, Rossi JE, Peng X, Lai M, et al Anti-NMDA-receptor encephalitis: case series and analysis of the effects of antibodies. Lancet Neurol. 2008;7(12):1091–8. 10.1016/S1474-4422(08)70224-2 18851928 PMC2607118

[6] Viaccoz A, Desestret V, Ducray F, Picard G, Cavillon G, Rogemond V, et al Clinical specificities of adult male patients with NMDA receptor antibodies encephalitis. Neurology. 2014;82(7):556–63. 10.1212/WNL.0000000000000126 24443452

[7] Irani SR, Bera K, Waters P, Zuliani L, Maxwell S, Zandi MS, et al N-methyl-D-aspartate antibody encephalitis: temporal progression of clinical and paraclinical observations in a predominantly non-paraneoplastic disorder of both sexes. Brain. 2010;133(Pt 6):1655–67. 20511282 10.1093/brain/awq113 PMC2877907

[8] Graus F, Saiz A, Lai M, Bruna J, Lopez F, Sabater L, et al Neuronal surface antigen antibodies in limbic encephalitis: clinical-immunologic associations. Neurology. 2008;71(12):930–6. 10.1212/01.wnl.0000325917.48466.55 18794496 PMC2586945

[9] Pruss H, Dalmau J, Harms L, Holtje M, Ahnert-Hilger G, Borowski K, et al Retrospective analysis of NMDA receptor antibodies in encephalitis of unknown origin. Neurology. 2010;75(19):1735–9. 10.1212/WNL.0b013e3181fc2a06 21060097

[10] Armangue T, Titulaer MJ, Malaga I, Bataller L, Gabilondo I, Graus F, et al Pediatric anti-N-methyl-D-aspartate receptor encephalitis-clinical analysis and novel findings in a series of 20 patients. J Pediatr. 2013;162(4):850–6 e2. 10.1016/j.jpeds.2012.10.011 23164315 PMC3582718

[11] Suleiman J, Wright S, Gill D, Brilot F, Waters P, Peacock K, et al Autoantibodies to neuronal antigens in children with new-onset seizures classified according to the revised ILAE organization of seizures and epilepsies. Epilepsia. 2013;54(12):2091–100. 10.1111/epi.12405 24151870

[12] Suleiman J, Brilot F, Lang B, Vincent A, Dale RC. Autoimmune epilepsy in children: case series and proposed guidelines for identification. Epilepsia. 2013;54(6):1036–45. 10.1111/epi.12142 23551014

[13] Hacohen Y, Wright S, Waters P, Agrawal S, Carr L, Cross H, et al Paediatric autoimmune encephalopathies: clinical features, laboratory investigations and outcomes in patients with or without antibodies to known central nervous system autoantigens. J Neurol Neurosur Ps. 2013;84(7):748–55. 10.1136/jnnp-2012-303807 23175854 PMC3686256

[14] Dale RC, Irani SR, Brilot F, Pillai S, Webster R, Gill D, et al N-methyl-D-aspartate receptor antibodies in pediatric dyskinetic encephalitis lethargica. Ann Neurol. 2009;66(5):704–9. 10.1002/ana.21807 19938173

[15] Wandinger KP, Saschenbrecker S, Stoecker W, Dalmau J. Anti-NMDA-receptor encephalitis: a severe, multistage, treatable disorder presenting with psychosis. J Neuroimmunol. 2011;231(1–2):86–91. 10.1016/j.jneuroim.2010.09.013 20951441

[16] Gresa-Arribas N, Titulaer MJ, Torrents A, Aguilar E, McCracken L, Leypoldt F, et al Antibody titres at diagnosis and during follow-up of anti-NMDA receptor encephalitis: a retrospective study. Lancet Neurol. 2014;13(2):167–77. 10.1016/S1474-4422(13)70282-5 24360484 PMC4006368

[17] Cull-Candy S, Brickley S, Farrant M. NMDA receptor subunits: diversity, development and disease. Curr Opin Neurobiol. 2001;11(3):327–35. 11399431 10.1016/s0959-4388(00)00215-4

[18] Cull-Candy SG, Leszkiewicz DN. Role of distinct NMDA receptor subtypes at central synapses. Science's STKE: signal transduction knowledge environment. 2004;2004(255):re16 15494561 10.1126/stke.2552004re16

[19] Gleichman AJ, Spruce LA, Dalmau J, Seeholzer SH, Lynch DR. Anti-NMDA receptor encephalitis antibody binding is dependent on amino acid identity of a small region within the GluN1 amino terminal domain. J Neurosci. 2012;32(32):11082–94. 10.1523/JNEUROSCI.0064-12.2012 22875940 PMC3430387

[20] Di Pauli F, Mader S, Rostasy K, Schanda K, Bajer-Kornek B, Ehling R, et al Temporal dynamics of anti-MOG antibodies in CNS demyelinating diseases. Clin Immunol. 2011;138(3):247–54. 10.1016/j.clim.2010.11.013 21169067

[21] Granerod J, Ambrose HE, Davies NW, Clewley JP, Walsh AL, Morgan D, et al Causes of encephalitis and differences in their clinical presentations in England: a multicentre, population-based prospective study. Lancet Infect Dis. 2010;10(12):835–44. 10.1016/S1473-3099(10)70222-X 20952256

[22] Gable MS, Sheriff H, Dalmau J, Tilley DH, Glaser CA. The frequency of autoimmune N-methyl-D-aspartate receptor encephalitis surpasses that of individual viral etiologies in young individuals enrolled in the California Encephalitis Project. Clin Infect Dis. 2012;54(7):899–904. 10.1093/cid/cir1038 22281844 PMC3297648

[23] Suh-Lailam BB, Haven TR, Copple SS, Knapp D, Jaskowski TD, Tebo AE. Anti-NMDA-receptor antibody encephalitis: performance evaluation and laboratory experience with the anti-NMDA-receptor IgG assay. Clin Chim Acta. 2013;421:1–6. 10.1016/j.cca.2013.02.010 23454475

[24] Dale RC, Merheb V, Pillai S, Wang D, Cantrill L, Murphy TK, et al Antibodies to surface dopamine-2 receptor in autoimmune movement and psychiatric disorders. Brain. 2012;135(Pt 11):3453–68. 23065479 10.1093/brain/aws256

[25] McIlhinney RA, Le Bourdelles B, Molnar E, Tricaud N, Streit P, Whiting PJ. Assembly intracellular targeting and cell surface expression of the human N-methyl-D-aspartate receptor subunits NR1a and NR2A in transfected cells. Neuropharmacology. 1998;37(10–11):1355–67. 9849671 10.1016/s0028-3908(98)00121-x

[26] Meddows E, Le Bourdelles B, Grimwood S, Wafford K, Sandhu S, Whiting P, et al Identification of molecular determinants that are important in the assembly of N-methyl-D-aspartate receptors. J Biol Chem. 2001;276(22):18795–803. 11279200 10.1074/jbc.M101382200

[27] Hughes EG, Peng X, Gleichman AJ, Lai M, Zhou L, Tsou R, et al Cellular and synaptic mechanisms of anti-NMDA receptor encephalitis. J Neurosci. 2010;30(17):5866–75. 10.1523/JNEUROSCI.0167-10.2010 20427647 PMC2868315

[28] Planaguma J, Leypoldt F, Mannara F, Gutierrez-Cuesta J, Martin-Garcia E, Aguilar E, et al Human N-methyl D-aspartate receptor antibodies alter memory and behaviour in mice. Brain. 2015;138(Pt 1):94–109. 25392198 10.1093/brain/awu310 PMC4285189

[29] Dalmau J, Lancaster E, Martinez-Hernandez E, Rosenfeld MR, Balice-Gordon R. Clinical experience and laboratory investigations in patients with anti-NMDAR encephalitis. Lancet Neurol. 2011;10(1):63–74. 10.1016/S1474-4422(10)70253-2 21163445 PMC3158385

[30] Dahm L, Ott C, Steiner J, Stepniak B, Teegen B, Saschenbrecker S, et al Seroprevalence of autoantibodies against brain antigens in health and disease. Ann Neurol. 2014;76(1):82–94. 10.1002/ana.24189 24853231

[31] Zandi MS, Paterson RW, Ellul MA, Jacobson L, Al-Diwani A, Jones JL, et al. Clinical relevance of serum antibodies to extracellular N-methyl-d-aspartate receptor epitopes. J Neurol Neurosurg Ps. 2014 Sep 22. pii: jnnp-2014-308736. 10.1136/jnnp-2014-308736 [Epub ahead of print] PMC6055984 25246644

