# Supplementary figures and images for: Comparison of Diagnostic Accuracy of Microscopy and Flow Cytometry in Evaluating N-Methyl-D-Aspartate Receptor Antibodies in Serum Using a Live Cell-Based Assay
Source: PLoS One. 2015 Mar 27;10(3):e0122037. doi: 10.1371/journal.pone.0122037 (PMC4376531; doi:10.1371/journal.pone.0122037)

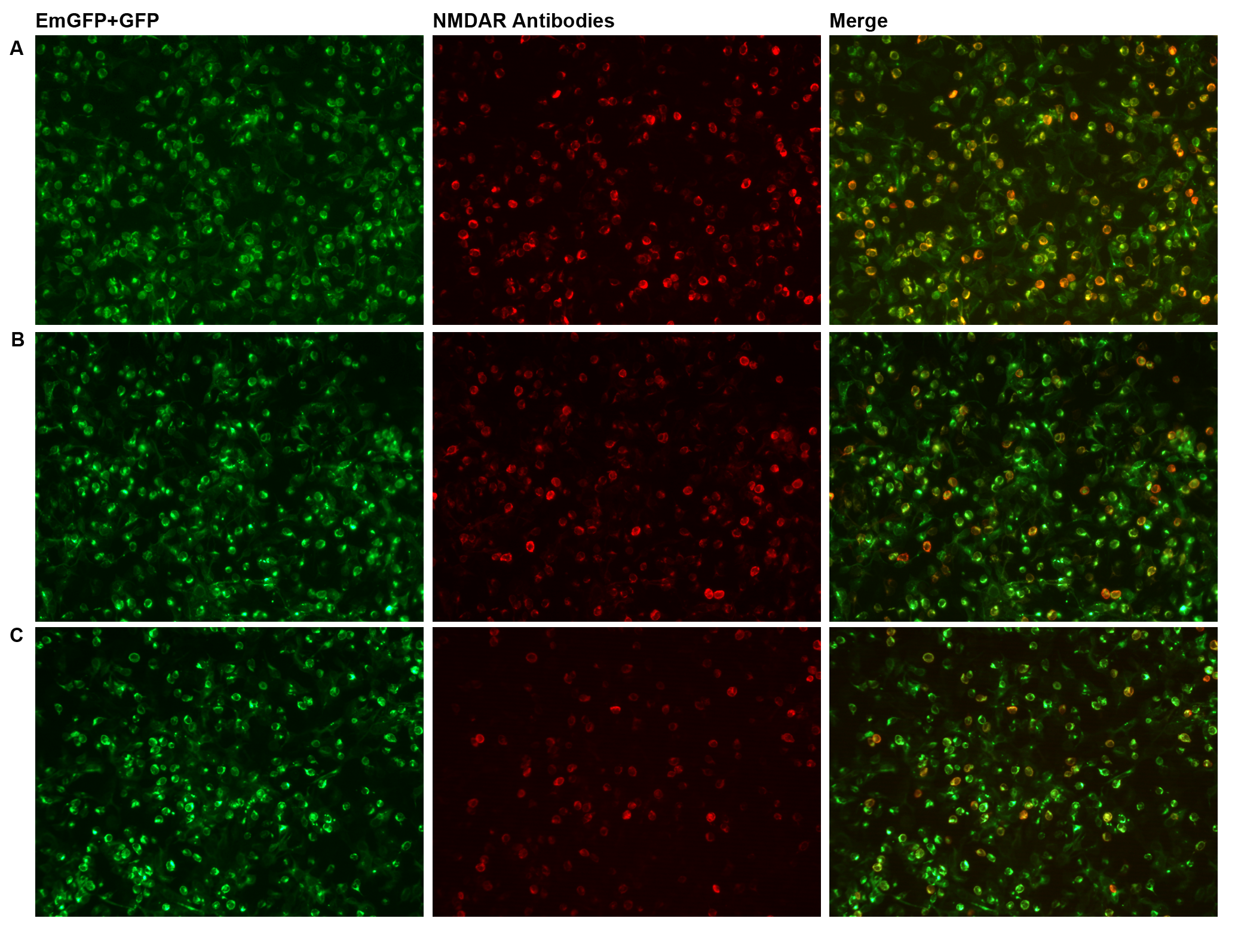

Supplement: S1 Fig — Cells stained with antibodies against NR1 (A), NR2A (B), and NR2B (C) are shown, respectively.(Em)GFP = (emerald) green fluorescent protein. NMDAR = N-methyl-D-aspartate receptor. (TIF) [file pone.0122037.s001.tif]

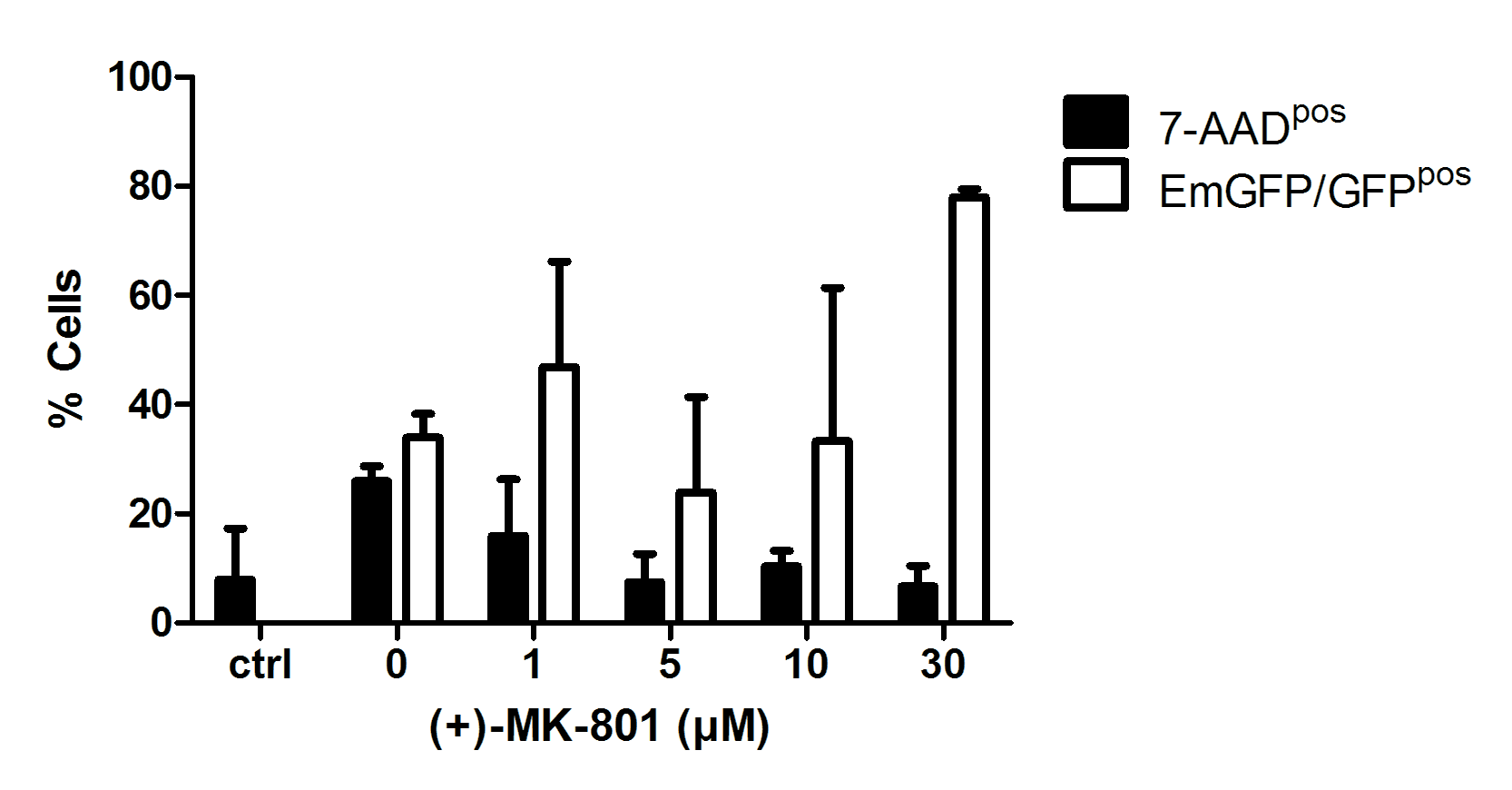

Supplement: S2 Fig — Dead cells are 7-AADpos, NMDAR expressing cells EmGFP/GFPpos. Means of two experiments are shown, bars indicate standard deviation.7-AAD = 7-amino-actinomycin D. (Em)GFP = (emerald) green fluorescent protein. NMDAR = N-methyl-D-aspartate receptor. (TIF) [file pone.0122037.s002.tif]

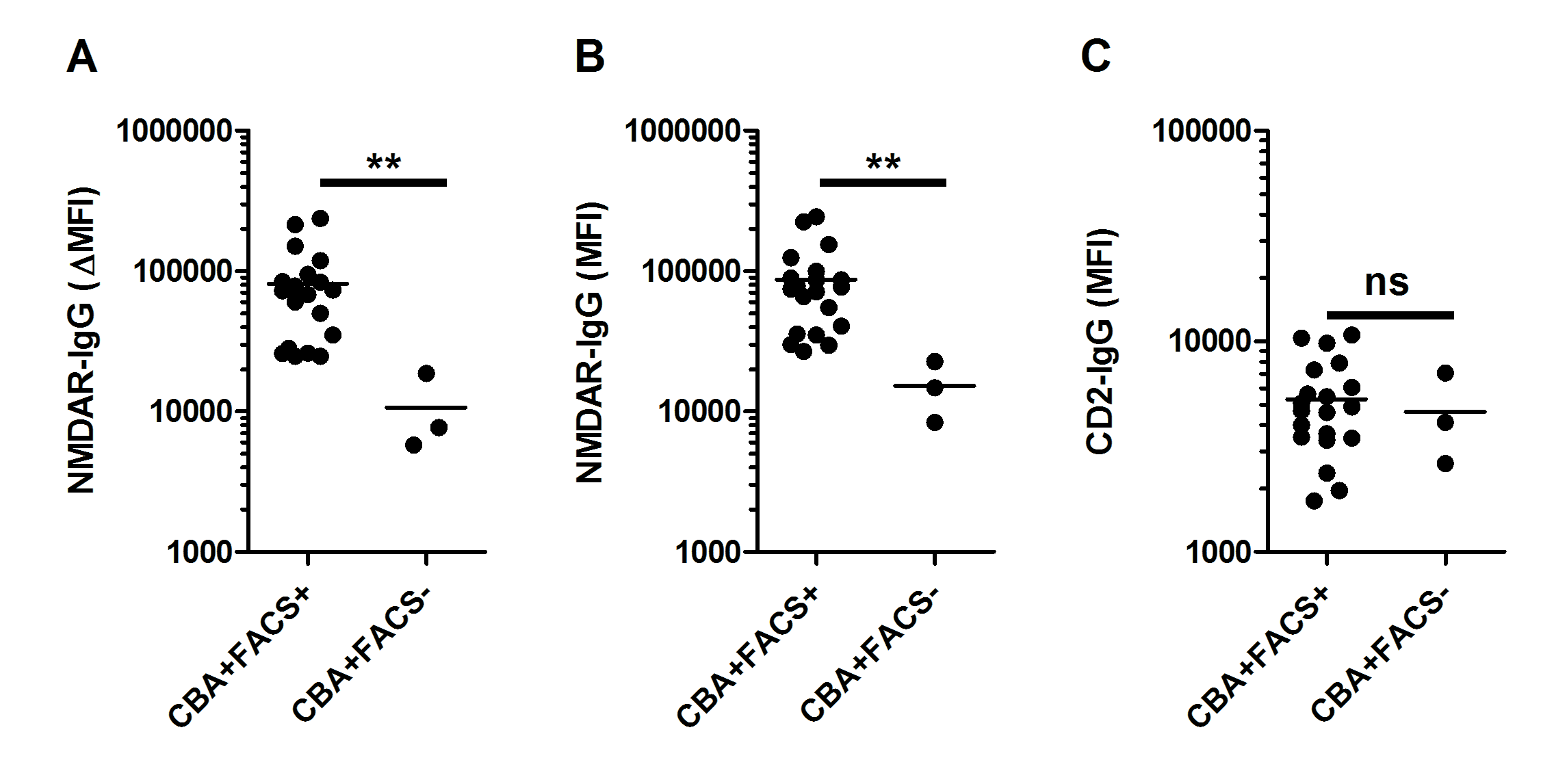

Supplement: S3 Fig — CBA+FACS+ show samples where NMDAR antibodies were detected with both methods, CBA+FACS- represent samples that were positive in the CBA, but (false) negative in the FACS. (A) ΔMFI (NMDAR-CD2 IgG). (B) MFI of IgG binding to NMDAR only. (C) MFI IgG binding to CD2 only (note that the scale of the y-axis has changed). Medians are indicated by horizontal bars. ΔMFI and MFI values were compared using a non-parametric test (Mann-Whitney U test). **p<0.01 CBA = cell-based assay. (Δ)MFI = (delta) median fluorescence intensity. FACS = fluorescence activated cell sorting. NMDAR = N-methyl-D-aspartate receptor. ns = not significant. (TIF) [file pone.0122037.s003.tif]

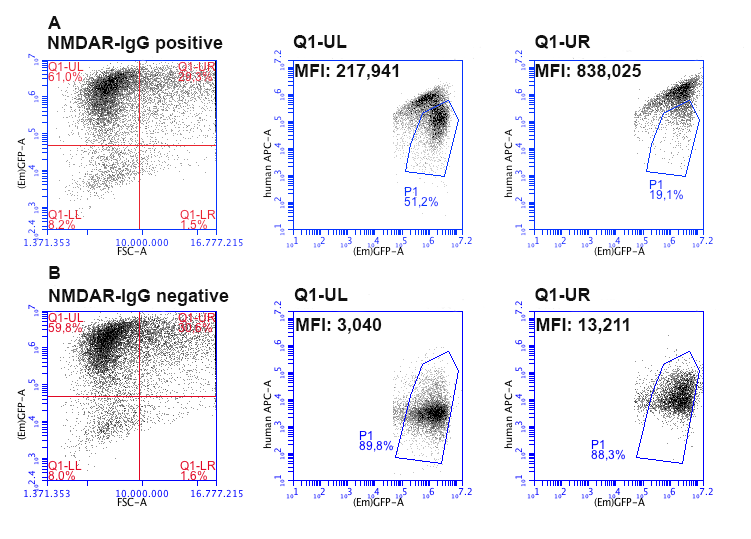

Supplement: S4 Fig — Left column: gating of (Em)GFP-positive NMDAR expressing HEK293A cells (excluding dead cells) to discriminate small (Q1-UL) and large (Q1-UR) cells. Middle column: relative APC fluorescence signal of small cells (Q1-UL). A second population with lower APC fluorescence signal is highlighted in blue (P1). Right column: relative APC fluorescence signal of large cells (Q1-UR). P1 decreased from 51.2% to 19.1% in the NMDAR-IgG positive sample. Overall MFI values are shown in the respective graphs. APC-A = allophycocyanin (area). (Em)GFP-A = (emerald) green fluorescent protein (area). FSC-A = forward scatter (area). MFI = median fluorescence intensity. NMDAR = N-methyl-D-aspartate receptor. Q1-UL/R = upper left/right quadrant. (TIF) [file pone.0122037.s004.tif]

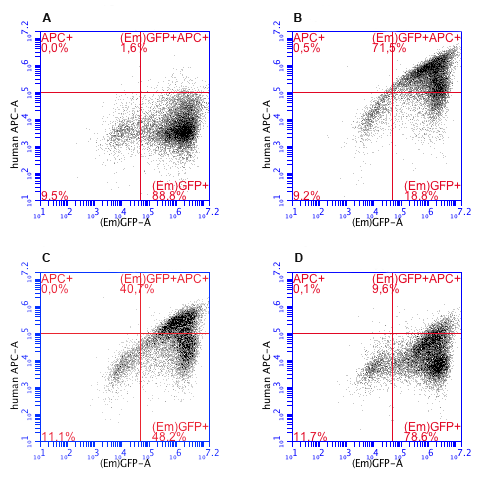

Supplement: S5 Fig — NMDAR transfected HEK293A cells (EmGFP/GFP positive) were incubated with human serum negative for NMDAR antibodies (A), or human serum high (B; CBA titer 1:20,480) and medium (C; CBA titer 1:640) positive for NMDAR antibodies which were detected by an APC-conjugated secondary antibody. The population within the upper right quadrant ((Em)GFPposAPCpos) represents the cell population expressing NMDAR with bound NMDAR antibodies. (D) shows the cells incubated with a serum negative in the FACS based assay, but positive in the CBA (1:640). Consider that positivity was not determined by the percentage of double positive cells, but the ΔMFI (A: -1,835; B: 290,060; C: 75,976; D: 8,160). APC-A = allophycocyanin (area). CBA = cell-based assay. ΔMFI = delta median fluorescence intensity. (Em)GFP(-A) = (emerald) green fluorescent protein (area). FACS = fluorescence activated cell sorting. NMDAR = N-methyl-D-aspartate receptor. (TIF) [file pone.0122037.s005.tif]

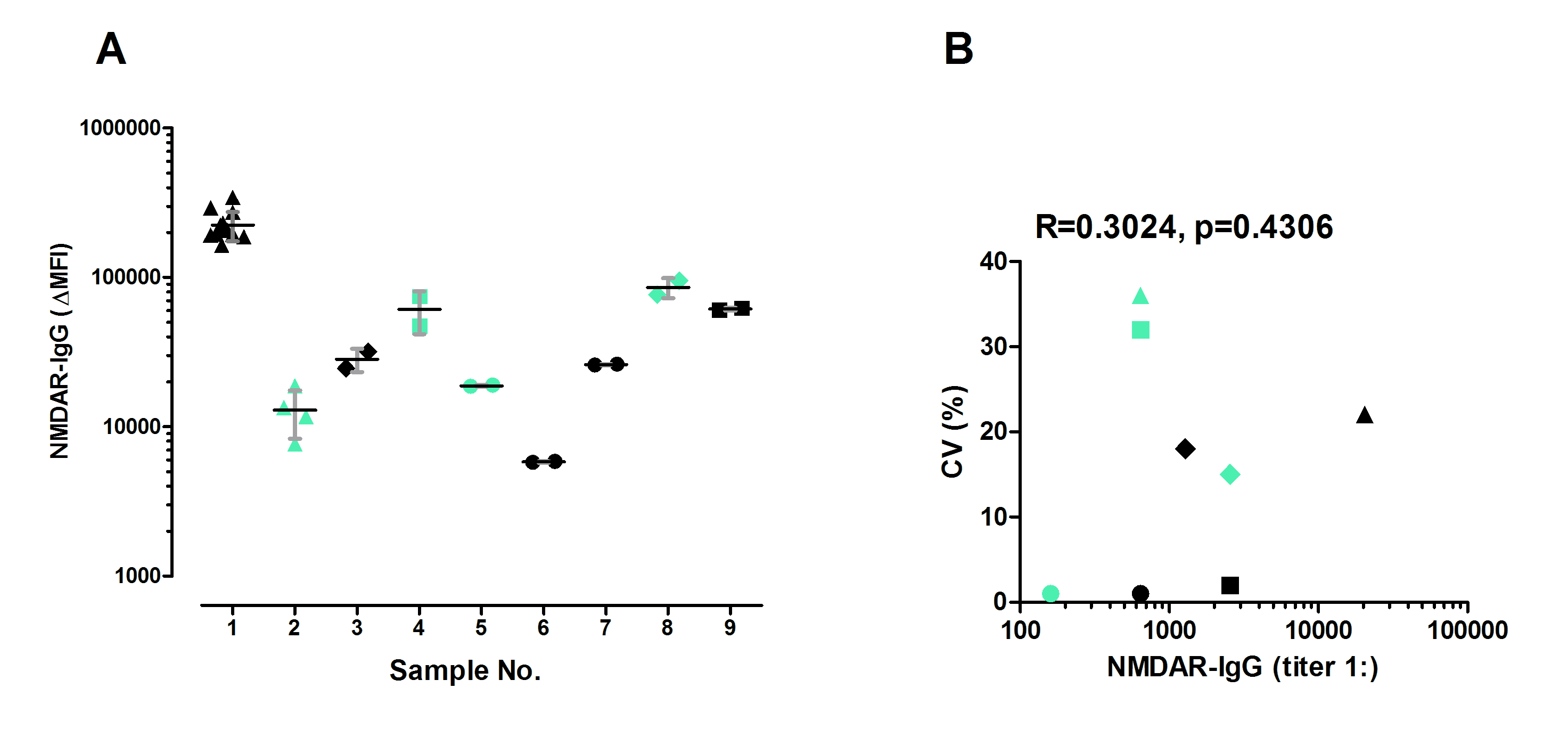

Supplement: S6 Fig — (A) Individual ΔMFI variability of the nine samples positive for NMDAR antibodies in the CBA. Means are shown as horizontal lines, standard deviations are indicated by grey bars. (B) Correlation of individual ΔMFI variability (CV) and respective NMDAR-IgG titers in the CBA. The correlation was calculated using non-parametric Spearman correlation. Correlation coefficient (R) and the p-value are shown in the graph. Symbols represent matching samples in (A) and (B). Sample Nos. 6 and 7 (A) have the same CV and NMDAR-IgG titer (B). CBA = cell-based assay. CV = coefficient of variation. ΔMFI = delta median fluorescence intensity. NMDAR = N-methyl-D-aspartate receptor. (TIF) [file pone.0122037.s006.tif]
